# Supplementary material for: Transcriptomic and Hormonal Changes in Wheat Roots Enhance Growth under Moderate Soil Drying
Source: Int J Mol Sci. 2024 Aug 23;25(17):9157. doi: 10.3390/ijms25179157 (PMC11395032; doi:10.3390/ijms25179157)
Supplement: Supplementary file 1 [file ijms-25-09157-s001.zip › ijms-3145771-supplementary.pdf]

Supplementary Materials

Figure S1. Effects of soil drying on wheat shoot fresh weight (A) and dry weight (B).

Figure S2.The distribution of gene expression levels in each sample.

Figure S3. The heatmap of differential gene clustering analysis between different samples.

Figure S4. The fitting results of standard curve of plant hormones in wheat roots under well-watered (WW) and soil drying (SD).

Table S1. The quality control of RNA-Seq data.

Table S2. Primary properties of topsoil (0-20 cm) at the test soil.

Table S3. Gene specific primers used in real-time RT-PCR.

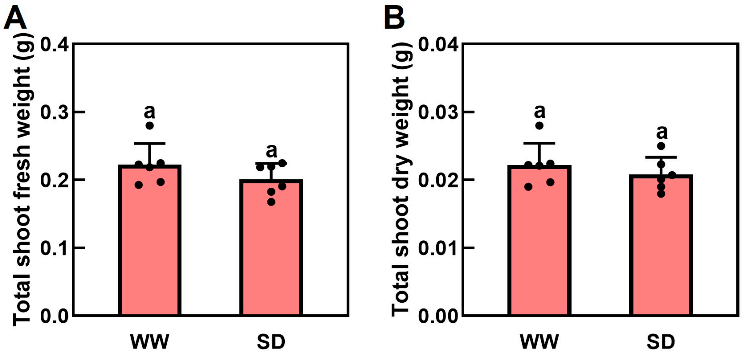

Figure S1. Effects of soil drying on wheat shoot fresh weight (A) and dry weight (B). The data in Figures are presented as mean ± standard error (n = 6). The same letter indicates no significant difference, while different letters indicate significant differences (*P* < 0.05, Duncan's test).

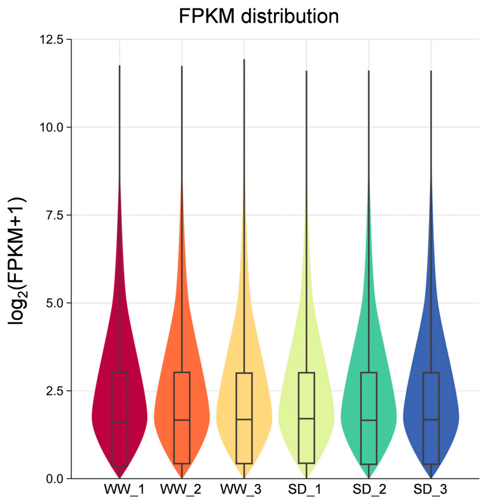

Figure S2. The distribution of gene expression levels in each sample. The horizontal axis is the name of the different samples, and the vertical axis is log2 (FPKM+1). FPKM: the number of transcripts per million mapped reads per thousand bases of transcription.

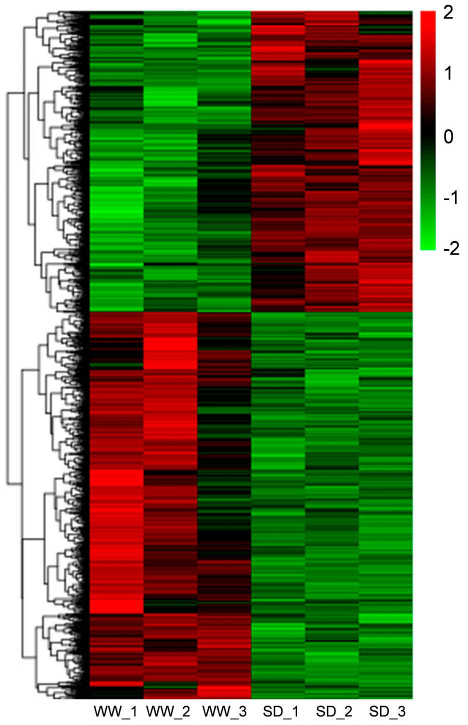

Figure S3. The heatmap of differential gene clustering analysis between different samples. The horizontal axis in the figure is the sample group. The name is the normalized value of the differential gene FPKM on the vertical axis. The redder the color, the higher the expression level, and the greener the color, the lower the expression level.

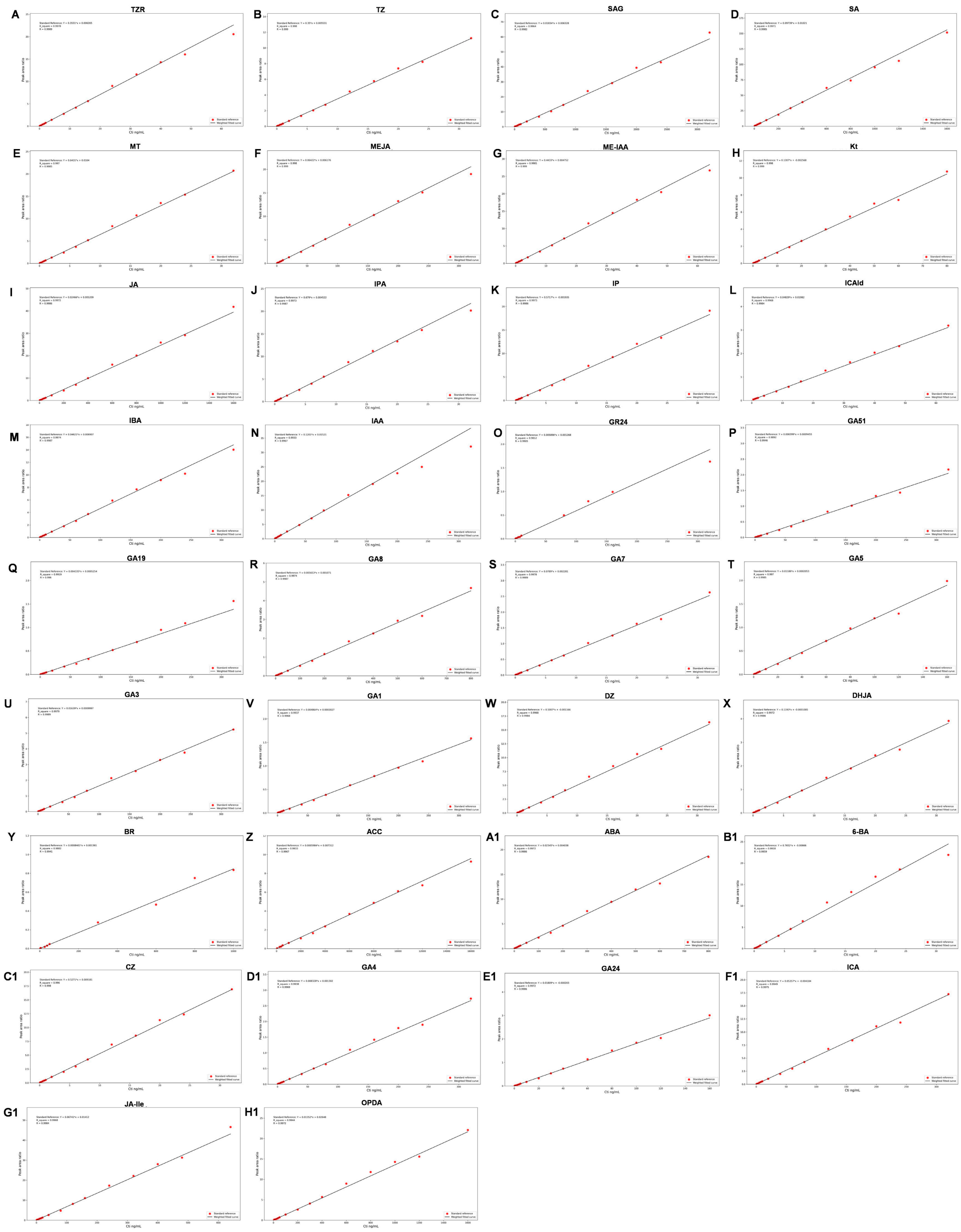

Figure S4. The fitting results of standard curve of plant hormones in wheat roots under well-watered (WW) and soil drying (SD). Gibberellin A (GA), Abscisic acid (ABA), Salicylic acid (SA), salicylic acid 2-O-β-D- glucose (SAG), Jasmonate acid (JA), 3-Indoleformic acid (ICA), Indole-3-acetic acid (IAA), 3-Indolebutyric acid (IBA), Cis-zeatin (CZ), Trans-zeatin riboside (TZR), N6-(delta 2-Isopentenyl)-adenine (IP), N6-isopentenyladenosine-D6 (IPA),

DL-dihydrozeatin (DZ), Benzyladenine (6-BA), 1-Aminocyclopropane-1-carboxylic acid (ACC), Dihydrojasmonicacid (DHJA), N-[(-)-jasmonoyl]-(S)-isoleucine (JA-Ile), Methyl 2-(1H-indol-3-yl)acetate (Me-IAA), Indole-3-carboxaldehyde (ICAId), Trans-Zeatin (TZ), Methyl jasmonate (MEJA), Brassinolide (BR), Melatonin (MT), Kinetin (Kt), 12-oxophytodienoic acid (OPDA).

Table S1. The quality control of RNA-Seq data.

Sample name	Raw reads	Raw bases	Clean reads	Clean bases	Error rate (%)	Q20 (%)	Q30 (%)	GC content (%)
WW_1	47836384	7.18G	45737326	6.86G	0.03	97.39	92.67	51.08
WW_2	43691946	6.55G	41997854	6.30G	0.03	97.4	93.04	53.08
WW_3	45596508	6.84G	44692322	6.70G	0.03	97.12	92.23	53.09
SD_1	47337542	7.10G	46356834	6.95G	0.03	96.94	91.81	53.41
SD_2	51414102	7.71G	50467776	7.57G	0.03	96.89	91.76	54.06
SD_3	44858294	6.73G	43906732	6.59G	0.03	97.1	92.19	53.61

Table S2. Primary properties of topsoil (0-20 cm) at the test soil.

pH	Organic matter ($g\ kg^{-1}$)	Total nitrogen ($g\ kg^{-1}$)	Alkali-hydrolyzable nitrogen ($mg\ kg^{-1}$)	Available phosphorus ($mg\ kg^{-1}$)	Available potassium ($mg\ kg^{-1}$)
6.15	26.38	1.60	164.13	29.33	141.25

Table S3. Gene specific primers used in real-time RT-PCR.

Gene	Primer (5' to 3')
Actin-6	[F]:CCAGTGGACGCACAACAGGTAT
	[R]:TCTTCATCAAACAGTCAGTTAGGTCG
TraesCS3A02G470800	[F]:TTCGATCAGTGAGATGCAGG
	[R]:CCCGCAACTTCTTCCTCCAA
TraesCS1B02G095900	[F]:CTTCCACAACTACCGCGTCT
	[R]:AGCCGAAGGTGATGAACTGG
TraesCS7D02G045700	[F]:CACCAAGCACTTGTTGTTCG
	[R]:GAGGTTCGATCAAGGAAGTATAATC
TraesCS2A02G099400	[F]:TCAACTCGGCCTCCTTCTGA
	[R]:AGCACCCAACCAGAAACGA
TraesCS2D02G098700	[F]:AGTCTGTCTCTTTGGGCTGC
	[R]:AGAAAGATGCACTGATTGGCG
TraesCS3A02G470700	[F]:GCCATCCCCAGATAGCAGAT
	[R]:AGTCCGCGATCACCATTGTT
